# A vital step determines the quality of human eggs: Spindle bipolarization

**DOI:** 10.1002/ctm2.70109

**Published:** 2024-11-28

**Authors:** Tianyu Wu, Yuxi Luo, Lei Wang, Qing Sang

**Affiliations:** ^1^ Institute of Pediatrics, Children's Hospital of Fudan University, State Key Laboratory of Genetic Engineering, Institutes of Biomedical Sciences, Shanghai Key Laboratory of Medical Epigenetics, Fudan University Shanghai China

1

The spindle bipolarity is essential for the accurate segregation of chromosomes into two distinct sets during cell division, encompassing both mitosis and meiosis.^1^ Reversal of spindle bipolarization typically leads to the formation of multipolar spindles, thereby resulting in defects in chromosome segregation.^2^ In mitosis, the bipolarization of the spindle relies on the paired centrosomes; supernumerary centrosomes resulting from centrosome overduplication or cytokinesis failure often lead to multipolarity of the spindle. The presence of multipolar spindles is frequently observed in human cancer cells and contributes to aberrant mitotic cell division.^3^ Similarly, abnormal spindle bipolarization in the human oocytes always leads to meiotic defects and aneuploid eggs,^4^ posing a threat to subsequent fertilization and early embryonic development. In contrast to mitosis, the centrosomes degenerate during oogenesis and are replaced by the human oocyte organizing centre (huoMTOC) for spindle microtubule polymerization.^5^ This suggests the existence of distinct mechanisms governing spindle bipolarity and multipolarity between human oocytes and mitotic cells. Therefore, it is imperative to investigate the mechanism underlying spindle bipolarization in human oocytes.

Recently, the process of spindle bipolarization in human oocytes was uncovered.^6^ The microtubules are nucleated from kinetochores, and the coalescence of microtubule minus ends in the kinetochore clusters is facilitated by the nuclear mitotic apparatus.^7^ This coalescence establishes the original spindle poles referred to as “minor poles”. Multiple minor poles are established during early prometaphase I due to the random distribution and clustering of kinetochores. Consequently, multipolar intermediates persist throughout prometaphase I. In contrast, mitotic cells or mouse oocytes do not exhibit multipolar intermediates since centrosomes or acentriole microtubule organizing centres (aMTOCs) are responsible for establishing bipolarity,^1^ respectively. The presence of prolonged multipolar intermediates involving minor poles represents a distinct phenotype exclusive to human oocytes in the absence of centrosomes or aMTOCs.

Essentially, spindle bipolarization encompasses three distinct phases, namely spindle microtubule amplification, spindle elongation, and regulation of spindle homeostasis. In order to elucidate the underlying mechanisms governing this process, a comprehensive analysis was conducted on 21 proteins associated with spindle bipolarity, revealing that only three of them—HAUS6, KIF11 and KIF18A—are indispensable for achieving proper spindle bipolarity. HAUS6 is essential for the initial phase of spindle bipolarization and responsible for microtubule‐dependent polymerization. In the subsequent phase, KIF11 facilitates the sorting and sliding of anti‐parallel microtubules, thereby promoting spindle elongation. Once spindle bipolarity is established, precise control over microtubule dynamics becomes crucial. Localization of KIF18A at kinetochores prevents excessive growth of kinetochore microtubules to ensure spindle stability. To assess the significance of spindle bipolarization in female meiosis, we conducted a screening for mutations in HAUS6, KIF11 and KIF18A among patients experiencing recurrent failure of in vitro fertilization (IVF) or intracytoplasmic sperm injection (ICSI). Remarkably, among 3627 infertile patients exhibiting abnormal oocyte maturation, fertilization, or early embryo development, we identified 11 individuals with mutations in HAUS6, KIF11 or KIF18A. Functional investigations revealed that these genetic alterations led to the impairment of spindle bipolarization and resulted in the formation of multipolar spindles. These findings unequivocally demonstrate the critical role of spindle bipolarization during human oocyte maturation and successful reproduction.

The egg with multiple polar bodies (Multi‐PB1s) before IVF or the zygote with multiple pronuclei (Multi‐PN) after ICSI are two novel phenotypes identified in the clinic. However, the underlying mechanisms remain unclear. The elucidation of the mechanism of human spindle bipolarization gives some clues for the generation of Multi‐PB1s and Multi‐PN. The generation of multipolar spindles plays a critical role (Figure 1). In the absence of centrosomes and aMTOCs, the minus ends of microtubules are coalesced to form multiple minor poles, forming typical mechanisms to establish the bipolar configuration in human oocytes. The defects of spindle bipolarity induced multipolar spindles, then cause multiple directional chromosome separations at anaphase I, therefore some chromosomes are ejected outwards to multiple first PB1s, while some chromosomes are ejected inwards to form multiple chromosome clusters that create Multi‐PN in the zygote. The Multi‐PB1s‐egg or Multi‐PN‐zygote finally causes abnormalities in oocyte maturation, fertilization, or embryonic development.

**FIGURE 1:**
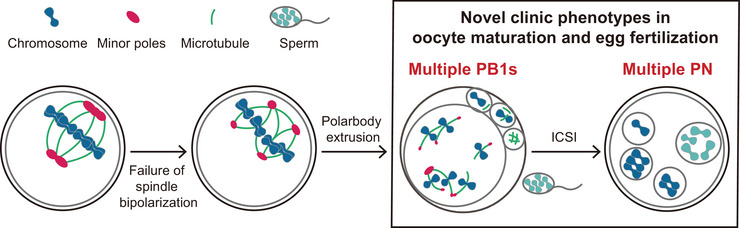
The present study investigates and observes two novel phenotypes resulting from abnormal spindle bipolarization during female meiosis I, providing further evidence to support the clinical diagnosis.

In summary, this investigation not only elucidates the molecular mechanism underlying spindle bipolarization but also provides insights into the pathological mechanisms of bipolarization defects. HAUS6, KIF11 or KIF18A could serve as precise genetic markers for diagnosing patients with Multi‐PB1s eggs or Multi‐PN zygotes. Furthermore, these genes could be potential molecular targets for exploring intervention strategies in future clinical applications.

## CONFLICT OF INTEREST STATEMENT

The authors declare no conflict of interest.
